# Nonlinear relationship between serum uric acid and body mass index: a cross-sectional study of a general population in coastal China

**DOI:** 10.1186/s12967-019-02142-9

**Published:** 2019-11-25

**Authors:** Hui Zhou, Zhen Liu, Zhong Chao, Yeqing Chao, Lidan Ma, Xiaoyu Cheng, Yangang Wang, Changgui Li, Ying Chen

**Affiliations:** 1 Department of Endocrinology and Metabolism, The Affiliated Hospital of Qingdao University, 16 Jiangsu Road, Qingdao, Shandong 266003 China; 2 Heze Medical College, Heze, Shandong China; 3 Shandong Provincial Key Laboratory of Metabolic Diseases, The Affiliated Hospital of Qingdao University, Qingdao, China

**Keywords:** Uric acid, Serum, Body mass index, Cross-sectional studies, Epidemiology, China

## Abstract

**Background:**

Conflicting evidence exists on the relationship between body mass index (BMI) and serum uric acid (SUA). Therefore, we aimed to evaluate the SUA–BMI relationship in a large-scale epidemiological survey in coastal China.

**Methods:**

This survey was conducted among the general population in the coastal region of China from September 2014 to January 2015. SUA Levels were measured by the automatic Sysmex Chemix-180 biochemical analyzer.

**Results:**

A total of 6098 men (BMI: 24.58 ± 3.74 kg/m^2^) and 7941 women (24.56 ± 3.64 kg/m^2^) were included in this study. A stronger positive BMI-SUA association was found for men than women (all *P*-values < 0.05). The piecewise linear spline models indicated a U-shaped relationship of SUA-BMI association for both men and women; and the lowest turning points were at 19.12 kg/m^2^ for men and 21.3 kg/m^2^ for women. When BMIs were lower than the nadir point, each 1 kg/m^2^ increase in BMI related to a 7.74-fold (95% CI − 14.73, − 0.75) reduction for men and 2.70-fold reduction (− 4.47, − 0.94) for women in SUA levels. Once the BMI was higher than the nadir point, each 1 kg/m^2^ increase in BMI was related to a 5.10-fold (4.44, 5.77) increment for men and 3.93-fold increment (3.42, 4.43) for women in SUA levels. The regression coefficient differences between the two stages were 12.84 (5.66, 20.03) for men and 6.63 (4.65, 8.61) for women.

**Conclusions:**

A U-shaped relationship between BMI and SUA was found for both men and women; the association was stronger for men than women.

## Background

As the end product of the metabolic breakdown of purine nucleotides [1], an increasing amount of research has indicated that the role of uric acid (UA) in metabolic syndrome has changed from innocent bystander to central player [2] and UA plays a key role in the development of hypertension [3, 4], hyperglycemia [5], hyperlipidemia [6], and also obesity [7]. Obesity, a multiple organ-system disease with underlying metabolic abnormalities, is a public health crisis and results in a huge economic burden [8, 9]. A series of cross-sectional studies suggested a positive association between serum uric acid (SUA) and body mass index (BMI) [10–13], which was demonstrated in a population-based longitudinal study [7]. However, limited data is available in the Chinese population.

In addition, several laboratory studies have suggested discrimination in the SUA-BMI association among different BMI levels [3, 14–16]. Our previous study also found that SUA and blood pressure (BP) might have a nonlinear, instead a simple linear, relationship [3]. Therefore, in this study, we used data from a large cross-sectional study in China to better understand the relationship between SUA and BMI, and additionally explore whether other metabolic factors may affect the relationship between SUA and BMI.

## Methods

### Study population

This is a population-based cross-sectional study among people in the coastal region of China, from August 2004 to December 2014. Participants were excluded if one of the following criteria were met: (I) medication use to lower weight, serum lipids, uric acid, blood sugar or blood pressure; (II) history of liver, severe renal, or heart diseases. A total of 14,039 participants (6098 males and 7941 females) met the criteria for enrollment in this study. The study protocol was approved by the ethics committee of the Affiliated Hospital of Qingdao University and informed consent was obtained from each participant.

### Data collection and measures

The participants’ demographic and lifestyle information were collected using a standard questionnaire by in-person interview, including current smoking status (smoker, never, past), alcohol consumption (never, moderate, heavy, past), and occupation type (light, moderate, heavy physical). BMI was calculated as weight (kg) per height squared (m^2^) and categorized as: under-weight (BMI < 18.5 kg/m^2^), normal weight (18.5 kg/m^2^ ≤ BMI < 24.9 kg/m^2^), overweight (25 kg/m^2^ ≤ BMI < 29.9 kg/m^2^), and obesity (BMI ≥ 30 kg/m^2^). For alcohol consumption in past 6 months, different from that used in the US and EU, heavy drinking was defined as equal as or greater than one time per week; and moderate drinking was defined as drinking at holiday and festival days, averagely one time per month. For occupation types, the “light physical” jobs referred to those with sedentary/desk job, such as official staffs, teachers and light physical houseworkers. Moderate physical work included students, gym teachers, and light physical farmworkers. And heavy physical works referred to the porter (workers who employed to help carry, ship or move luggage or other loads), construction workers, athletes, and so on. BP was measured with a standard mercury sphygmomanometer, and subjects were required to rest for at least 15 min before BP measurement. All measurements of height, weight, and BP were carried out by the same group of seven professional physicians.

Elbow venous blood (5 mL) was extracted from all participants after fasting for at least 12 h. The fasting blood glucose (FBG), triglyceride (TG), total cholesterol (TC), creatinine, high/low-density lipoprotein cholesterol (H/LDL), and SUA levels of all blood specimens were examined by automatic Sysmex Chemix-180 biochemical analyzer (Nanchang Micare Medical Equipment Co., LTD, Jiangxi, China). Estimated glomerular filtration rate (eGFR) was calculated by the following formula: eGFR = 175 × (creatinine/88.4)^−1.234^ × age^−0.179^ × (0.79 for females). Hyperuricemia was diagnosed if SUA levels were higher than 420 µmol/L for men and postmenopausal women, and higher than 357 µmol/L for premenopausal women.

### Statistical analyses

Previous studies suggested large differences in BMI and SUA among men and women; therefore, all analyses were separately applied to men and women. We calculated mean ± standard deviation (SD) and median (interquartile range) for frequency of participant characteristics, t-tests for normal distributions, Kruskal–Wallis tests for non-normal distributions, and Chi square tests were used to compare characteristic differences among men and women. We evaluated the possible linear and nonlinear relationships between SUA and BMI by multivariate linear regression models and two-piece piecewise regression models adjusted for age, current alcohol consumption status, current smoking status, occupation type, systolic blood pressure (SBP), diastolic blood pressure (DBP), FBS (log transformed), eGFR, low density lipoprotein (LDL), and TC, among men and women. We further conducted stratified and interaction analyses to explore the potential modifier and interaction effects on the SUA-BMI association. Before these, covariate screening was also performed among all variables included in Table 1 using univariate analysis. 93.7% of the participants enrolled in our study had complete data, and only those participants with complete data were included in the analysis.

**Table 1:** Characteristics of 7941 women and 6098 men included in this study

Characteristics	Men	Women	Total	P-value
n	6098	7941	14,039	
Hyperuricemia	17.96%	4.04%	10.18%	< 0.001
Age (years)	47.70 ± 14.28	48.65 ± 13.65	48.24 ± 13.94	< 0.001
Body mass index (kg/m^2^)	24.54 ± 3.51	24.58 ± 3.74	24.56 ± 3.64	0.535
Occupation types				< 0.001
Light physical	3266 (53.56)	5606 (70.60)	8872 (63.20)	
Moderate physical	2123 (34.81)	1901 (23.94)	4024 (28.66)	
Heavy physical	709 (11.63)	434 (5.47)	1143 (8.14)	
Serum uric acid (μmol/L)	349.62 ± 85.30	267.37 ± 70.79	303.09 ± 87.50	< 0.001
Smoking status in last 6 months (%)				< 0.001
Smoking	2804 (45.98)	7769 (97.83)	10,573 (75.31)	
Never	2972 (48.74)	152 (1.91)	3124 (22.25)	
Past	322 (5.28)	20 (0.25)	342 (2.44)	
Alcohol drinking status in last 6 months (%)				< 0.001
Never	2659 (43.60)	7698 (96.94)	10,357(73.77)	
Moderate	2204 (36.14)	180 (2.27)	2384 (16.98)	
Heavy	1161 (19.04)	62 (0.78)	1223 (8.71)	
Quit	74 (1.21)	1 (0.01)	75 (0.53)	
Systolic blood pressure (mmHg)	131.76 ± 19.38	129.73 ± 22.13	130.61 ± 21.01	< 0.001
Diastolic blood pressure (mmHg)	85.68 ± 12.07	82.49 ± 11.82	83.87 ± 12.03	< 0.001
Fasting blood glucose (mmol/L)	5.10 (4.42-5.70)	5.08 (4.50-5.68)	5.09 (4.48–5.70)	0.362
Triglyceride (mmol/L)	1.20 (0.82–1.90)	1.10 (0.74–1.66)	1.14 (0.77–1.76)	< 0.001
High-density lipoprotein cholesterol (mmol/L)	1.31 ± 0.42	1.40 ± 0.38	1.36 ± 0.40	< 0.001
Low-density lipoprotein cholesterol (mmol/L)	2.76 ± 0.82	2.74 ± 0.84	2.75 ± 0.83	0.330
Total cholesterol (mmol/L)	4.86 ± 1.04	4.88 ± 1.09	4.87 ± 1.07	0.277
Creatinine (μmol/L)	82.98 ± 21.77	70.11 ± 21.07	75.70 ± 22.31	< 0.001
Estimated glomerular filtration rate (mL/min/1.73 m^2^)	90.54 (76.98–126.17)	85.45 (73.57–127.39)	89.15 (75.28–136.30)	< 0.001

All statistical analyses were performed using Empower Stats software (X&Y Solutions, Inc., Boston, USA). *P* < 0.05 was considered statistically significant.

## Results

### Characteristics of the participants

A total of 14,039 participants aged 48.24 ± 13.94 years, of which 56.6% were women, met the criteria for enrollment and were included in the analyses. The overall mean level of SUA was 303.09 (± 87.50) μmol/L, which was significantly higher among men (349.62 ± 85.30 μmol/L) than women (267.37 ± 70.79 μmol/L). Hyperuricemia was diagnosed in 10% of participants, and hyperuricemia prevalence was significantly lower among women (4.04%) than men (17.96%) (Table 1).

After stratification by BMI, SUA levels increased from 273.62 ± 72.84 μmol/L for under-weight participants to 341.60 ± 93.59 μmol/L for obese participants (Table 2). Also, obese participants tended to be older, with less healthy lifestyles, and higher SBP, DBP, fasting glucose and cholesterol levels (all *P*-values < 0.05).

**Table 2:** Characteristics of 7941 women and 6098 men among 4 BMI categories in this study

Characteristics	Under-weight	Normal weight	Overweight	Obesity	P-value
n	470	7548	4983	1038	
Hyperuricemia	4.81%	7.19%	13.46%	17.68%	< 0.001
Age (years)	42.49 ± 18.60	46.64 ± 14.28	50.42 ± 12.51	51.98 ± 12.87	< 0.001
Body mass index (kg/m^2^)	17.59 ± 0.75	22.33 ± 1.73	27.06 ± 1.36	32.01 ± 2.21	< 0.001
Occupation types, %					< 0.001
Light physical	290 (61.70)	4555 (60.35)	3294 (66.10)	733 (70.62)	
Moderate physical	147 (31.28)	2322 (30.76)	1311 (26.31)	244 (23.51)	
Heavy physical	33 (7.02)	671 (8.89)	378 (7.59)	61 (5.88)	
Serum uric acid (μmol/L)	273.62 ± 72.84	287.58 ± 82.84	321.35 ± 88.14	341.60 ± 93.59	< 0.001
Smoking status in last 6 months (%)					< 0.001
Smoking	354 (75.32)	5575 (73.86)	3816 (76.58)	828 (79.77)	
Never	110 (23.40)	1792 (23.74)	1031 (20.69)	191 (18.40)	
Past	6 (1.28)	181 (2.40)	136 (2.73)	19 (1.83)	
Alcohol drinking status in last 6 months (%)					< 0.001
Never	373 (79.36)	5589 (74.05)	3588 (72.00)	807 (77.75)	
Moderate	56 (11.91)	1259 (16.68)	911 (18.28)	158 (15.22)	
Heavy	38 (8.09)	657 (8.70)	457 (9.17)	71 (6.84)	
Quit	3 (0.64)	43 (0.57)	27 (0.54)	2 (0.19)	
Systolic blood pressure (mmHg)	118.08 ± 17.73	126.39 ± 19.85	135.75 ± 20.41	142.34 ± 22.28	< 0.001
Diastolic blood pressure (mmHg)	76.57 ± 10.21	81.39 ± 11.15	86.86 ± 11.90	90.94 ± 12.85	< 0.001
Fasting blood glucose (mmol/L)	4.70 (4.10–5.20)	4.92 (4.40–5.50)	5.20 (4.60–5.88)	5.60 (4.90–6.31)	< 0.001
Triglyceride (mmol/L)	0.78 (0.59–1.07)	0.97 (0.68–1.44)	1.41 (0.96–2.14)	1.70 (1.18–2.57)	< 0.001
High-density lipoprotein cholesterol (mmol/L)	1.47 ± 0.35	1.41 ± 0.41	1.30 ± 0.38	1.27 ± 0.38	< 0.001
Low-density lipoprotein cholesterol (mmol/L)	2.39 ± 0.73	2.65 ± 0.81	2.89 ± 0.83	2.95 ± 0.80	< 0.001
Total cholesterol (mmol/L)	4.42 ± 0.96	4.73 ± 1.06	5.04 ± 1.05	5.21 ± 1.03	< 0.001
Creatinine (μmol/L)	71.03 ± 21.83	74.47 ± 23.02	77.37 ± 21.26	78.73 ± 21.23	< 0.001
Estimated glomerular filtration rate (%)	99.92 (80.05–158.5)	91.35 (76.59–142.9)	86.36 (73.56–126.8)	83.69 (72.03–113.2)	< 0.001

### Linear relationship between SUA and BMI

The linear regression models suggested a significant association between SUA and BMI in both men and women, after adjustment for age, current smoking status, current alcohol consumption status, occupation types, SBP, DBP, FBS, LDL, TG, TC, and eGFR (all *P*-values < 0.05). In women, SUA levels were significantly increased by 3.03 µmol/L (95% CI 2.60, 3.46 µmol/L) for each SD (3.74 kg/m^2^) increase in BMI. Similar results were observed for men where SUA levels were elevated by 4.71 µmol/L (95% CI 4.08, 5.35 µmol/L) for each SD (3.51 kg/m^2^) increase in BMI (Table 3).

**Table 3:** Multivariable linear and non-linear relationship between sUA and BMI stratified by gender, *β* (95% CI) of BMI (kg/m^2^)

Models	Men	P-value	Women	P-value	Total	P-value
Linear regression model, Per SD increase in BMI						
Crude model	6.11 (5.56, 6.66)	< 0.0001	5.03 (4.66, 5.40)	< 0.0001	5.47 (5.15, 5.78)	< 0.0001
Model I	6.16 (5.60, 6.71)	< 0.0001	4.09 (3.71, 4.48)	< 0.0001	5.29 (4.97, 5.61)	< 0.0001
Model II	4.71 (4.08, 5.35)	< 0.0001	3.03 (2.60, 3.46)	< 0.0001	3.80 (3.44, 4.17)	< 0.0001
Non-linear model, regression coefficients (*β*)						
Break point of BMI, kg/m^2^ (K)	19.1		21.3		19.2	
< K	− 7.74 (− 14.7, − 0.75)	0.0301	− 2.70 (− 4.47, − 0.94)	0.0027	− 8.72 (− 12.4, − 5.06)	< 0.0001
≥ K	5.10 (4.44, 5.77)	< 0.0001	3.93 (3.42, 4.43)	< 0.0001	4.26 (3.87, 4.65)	< 0.0001
Difference of *β*-value between strata	12.8 (5.66, 20.0)	0.0005	6.63 (4.65, 8.61)	< 0.0001	13.0 (9.21, 16.8)	< 0.0001
Predicted value of sUA at break point	308 (304, 312)		243 (240, 245)		267 (264, 270)	
P-value for likelihood ratio test	< 0.001		< 0.001		< 0.001	

Model I: Adjusted for age

Model II: Adjusted for age, current smoking status, current drinking status, occupational types, SBP, DBP, fasting blood sugar (log_10_ transformed), eGFR, LDL, triglyceride (log_10_ transformed), and total cholesterol

Non-linear model: Adjusted for age, current drinking status, current smoking status, occupational types, SBP, DBP, fasting blood sugar (log_10_ transformed), eGFR, LDL, triglyceride (log_10_ transformed), and total cholesterol

### Nonlinear relationship between SUA and BMI

**Fig. 1:**
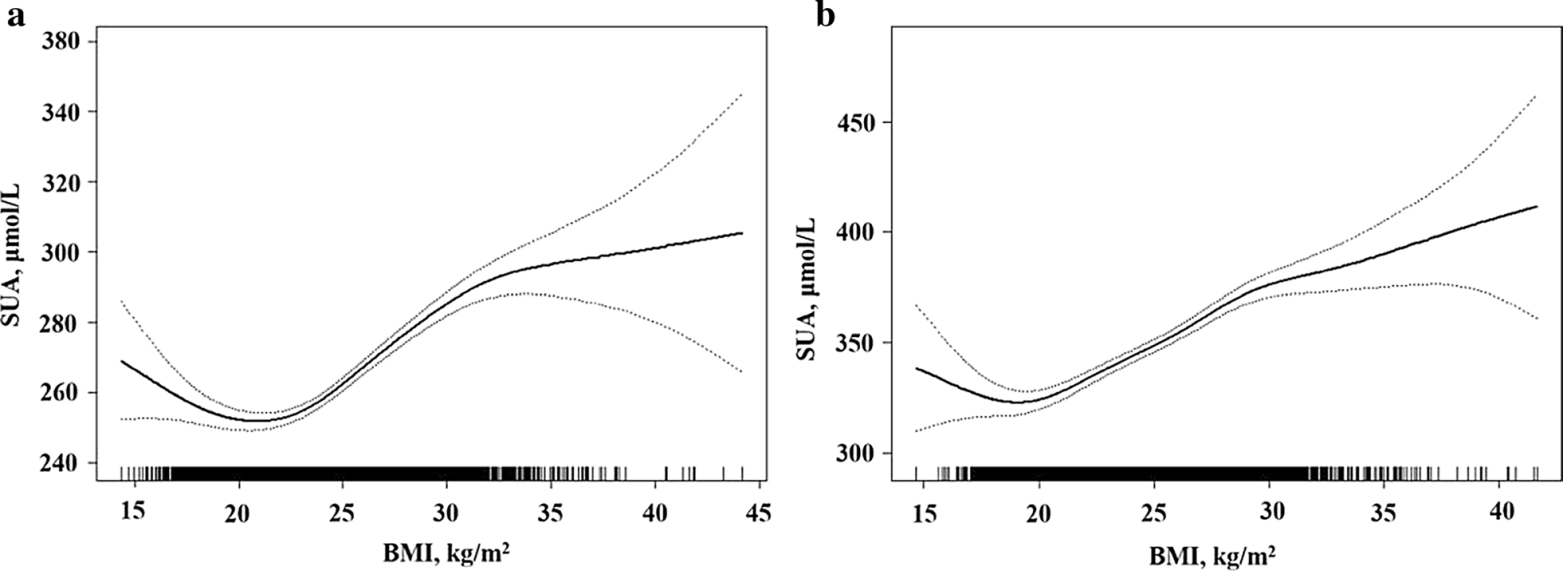
Two-piece piecewise regression and smooth curve-fitting for association between SUA and BMI stratified by gender. **a** The two-piece wise smooth curve for BMI-SUA association in women. When the BMI was smaller or equal than 21.3 kg/m^2^, there is a negative association between BMI and SUA. The regression coefficient *β* was − 2.70 (95% CI − 4.47, − 0.94). If BMI greater than 21.3 kg/m^2^, a significant positive association was found with a regression coefficient of 3.93 (95% CI 3.42, 4.43). **b** The two-piece wise smooth curve for BMI-SUA association in women. When BMI smaller or equal to 19.1 kg/m^2^, a negative BMI-SUA association was found with a regression coefficient of − 7.74 (95% CI − 14.7, − 0.75). When BMI greater than 19.1 kg/m^2^, a positive association was found, the coefficient was 5.10 (95% CI 4.44, 5.77). The two-piece wise models adjusted for age, current smoking status, current drinking status, occupational types, SBP, DBP, fasting blood sugar (log_10_ transformed), eGFR, LDL, triglyceride (log_10_ transformed), and total cholesterol

A U-shaped relationship between SUA and BMI was observed for both women and men by piecewise regression model; and the estimated nadir point was 19.1 kg/m^2^ for men and 21.3 kg/m^2^ for women (Fig. 1 and Table 3). For men, once the BMI was lower than 19.1 kg/m^2^, a significantly positive association between BMI and SUA was found, and the regression coefficient was − 7.74 (95% CI − 14.7, − 0.75; *P *= 0.03) per SD increase in BMI; while a negative BMI-SUA association was detected if the BMI was higher than 19.1 kg/m^2^, and the regression coefficient was 5.10 (95% CI 4.44, 5.77; *P* < 0.0001) per SD increase in BMI. In women, SUA levels were negatively associated with BMI if the BMI was lower than 21.3 kg/m^2^, and the regression coefficient was − 2.70 (95% CI − 4.47, − 0.94; *P* = 0.003) per SD increase in BMI; then when the BMI was higher than 21.3 km/m^2^, the SUA levels positively increased with BMI elevation, and the estimated regression coefficient was 3.93 (95% CI 3.42, 4.43; *P *< 0.0001) per SD increase in BMI.

The calculated differences in regression coefficients higher and lower than the nadir point were 12.8 (95% CI 5.66, 20.0; *P *= 0.0005) for men and 6.63 (95% CI 4.65, 8.61; *P *< 0.0001) for women, after adjustment for age, current smoking and alcohol consumption status, SBP, DBP, TC, TG, FBS, and eGFR.

### Exploration of modifier and interaction effects on SUA-BMI-association

We explored potential modifier or interaction effects from TG, TC, FBS, and LDL, and found that TG might be a potential interaction factor for SUA-BMI association (Table 4). After stratified analyses by tertiles of TG levels, we found the SUA-BMI association (regression coefficients per SD increase in BMI) among men was significantly increased from 3.74 (95% CI of regression coefficient: 2.59, 4.90) at tertile 1 (< 14.4 µmol/L), to 4.89 (95% CI 3.84, 5.95) at tertile 2 (14.5–22.9 µmol/L), and to 6.09 (95% CI 5.13, 7.05) at tertile 3 (≥ 22.9 µmol/L) with *P* value of 0.002 for linear trend and *P*-value of 0.007 for interaction. Among women, a significant TG interaction was detected. The SUA-BMI regression coefficients were 2.12 (95% CI 1.41, 2.83) for tertile 1, 3.29 (95% CI 2.61, 3.97) for tertile 2, and 4.25 (95% CI 3.51, 4.99) for tertile 3 with *P* < 0.0001 for linear trend and *P *=0.0002 for interaction.

**Table 4:** Potential interactions of triglyceride with sUA-BMI associations among men and women

Models	Regression coefficients (95% CI) for triglyceride			P for interaction	P for linear trend
	T1: 0.09–0.81, mmol/L	T2: 0.81–1.21, mmol/L	T3:1.21–15.87, mmol/L		
Men	3.74 (2.59, 4.90)	4.89 (3.84, 5.95)	6.09 (5.13, 7.05)	0.0074	0.0017
Women	2.12 (1.41, 2.83)	3.29 (2.61, 3.97)	4.25 (3.51, 4.99)	0.0002	< 0.0001
Total	2.91 (2.28, 3.54)	4.03 (3.44, 4.62)	5.20 (4.60, 5.80)	< 0.0001	< 0.0001

Adjusted for age, current smoking status, alcohol consumption, occupational types, SBP, DBP, fasting blood sugar (log_10_ transformed), eGFR, LDL, triglyceride (log_10_ transformed) and total cholesterol

## Discussion

Positive association between SUA and BMI was found among men and women. Using the two-piece piece-wise regression model, we found a U-shaped SUA-BMI relationship for both men and women. Although a positive association was maintained when the BMI was higher than 20 kg/m^2^, a negative association was found when the BMI was lower than 20 kg/m^2^. Our results suggested that SUA level might be a good index for BMI, depending on whether or not the participants were underweight.

Accumulating evidence suggests that elevated SUA levels are common comorbidities of obesity and are accompanied by gradually increasing BMI [17]. Dr. Rathmann and colleagues used the data from the Coronary Artery Risk Development in Young Adults (CARDIA) Study (1249 male and 1362 female black and white subjects aged 17–35 years with a 10-year follow-up) to evaluate changes in SUA with changes in components of the metabolic syndrome in young adults [18]. They found that BMI had a significant independent linear association with UA in all race-sex-groups. Another Chinese study of 2962 patients with type 2 diabetes also observed a linear association between the prevalence of obesity and increasing SUA levels [19]. However, these studies only focused on the linear relationship between SUA and BMI, while our results indicated a strong U-shaped relationship for both men and women. Previous mechanism studies suggested that chronic inflammation contributed to the pathogenesis of obesity [20, 21]; the infiltration and accumulation of macrophages in adipose tissue was demonstrated to be associated with increased tumor necrosis factor-α and interleukin 6 secretion [22, 23]. Similarly, macrophages also play an important role in the inflammation associated with gout/hyperuricemia [24–27]. Of note, physiological concentrations of SUA displayed anti-inflammatory effects both in vitro and in vivo [14] and thus might partly explain the U-shaped relationship between SUA and BMI.

Meanwhile, our results also showed a similar U-shaped SUA-BMI association and nadir point of BMI, around 20 kg/m^2^, for men and women. The strength of the association was significantly stronger for men than women. A prospective study on 3857 Chinese participants with normal metabolic function [28] found that baseline SUA levels and metabolic syndrome during a mean follow-up of 5.41 years were more closely related in women than in men. A study among Mexican-origin infants, youth and adults [10] also found a stronger association between salivary UA levels and BMI for females than males. Although the mechanisms underlying these observations are still unclear, several studies indicated that differences in sex hormone levels may partially explain the effects. An analysis study on the data from 7662 women aged 20 years and older in the Third National Health and Nutrition Examination Survey (1988–1994) found that menopause was independently associated with higher SUA levels, and postmenopausal hormone use was associated with lower UA levels among postmenopausal women [29]. A study on 128 obese patients who underwent laparoscopic sleeve gastrectomy found that increased estradiol levels, decreased total testosterone levels, and increased estradiol/total testosterone ratios in obese female patients 6 months post-surgery might be related to SUA improvement [30]. Additionally, mechanism-related evidence also suggested that estradiol can affect fat metabolism and distribution in women.

In addition, we found an interaction effect of TG levels on the association between BMI and SUA; BMI had a stronger effect on SUA at higher TG levels. Similar interdependent relationships have been reported among SUA, C-reactive protein and interleukin 10 levels, which related to early hepatic damage [25]. As the hepatic manifestation of obesity and metabolic dysfunction, nonalcoholic fatty liver disease could result in hepatic damage. However, these assumptions need further investigation in additional well-designed studies.

Our study has several limitations. First, due to the inherent nature of cross-sectional designs, our results could not make a causality conclusion. Second, the participants were limited to coastal areas and had special diet features with more marine food products, such as sea-fish, shrimp, and shellfishes. Therefore the extension of these conclusions should be prudent. Furthermore, additional large-scale studies with representative populations are warranted to validate our conclusions.

## Conclusions

Using the data from a general Chinese population, we found a U-shaped relationship between BMI and SUA for both men and women; and a stronger SUA-BMI association was found for men than for women. Further well-designed, large-scale longitudinal studies are needed to confirm our conclusions and evaluate the underlying mechanisms of the association.

## Data Availability

All data generated or analyzed during this study are included in this published article.

## Abbreviations

BMI: body mass index
BP: blood pressure
DBP: diastolic blood pressure
eGFR: estimated glomerular filtration rate
FBG: fasting blood glucose
H/LDL: high/low-density lipoprotein cholesterol
LDL: low density lipoprotein
SBP: systolic blood pressure
SD: standard deviation
SUA: serum uric acid
TC: total cholesterol
TG: triglyceride
UA: uric acid
